# Assessment of the risk of developing breast cancer using the Gail model in Asian females: A systematic review

**DOI:** 10.1016/j.heliyon.2020.e03794

**Published:** 2020-04-22

**Authors:** Solikhah Solikhah, Sitti Nurdjannah

**Affiliations:** a Faculty of Public Health, Universitas Ahmad Dahlan, Yogyakarta, 55166, Indonesia; b Dynamic Social Study Center, Universitas Ahmad Dahlan, Yogyakarta, 55166, Indonesia

**Keywords:** Cancer research, Health sciences, Public health, Epidemiology, Women's health, Breast cancer risk, Gail model, Systematic review

## Abstract

**Introduction:**

Currently, the Breast Cancer Risk Assessment Tool (BCRAT), also known as the Gail model (GM) has been widely recognized and adapted for to study disparity in racial and ethnic groups in America including Asian and Pacific Islander American females. However, its applicability outside America remains uncertain due to diversity in epidemiology and risk factors of breast cancer in populations especially in Asian females. We sought to evaluate the performance of the GM to predict breast cancer risk in Asian countries.

**Material and methods:**

This study identified articles published from 2010 by searching PubMed, MEDLINE, Scopus, Web of Science, Google Scholar and gray literature. The initial search terms were breast *cancer, mammary, carcinoma, tumor, neoplasm, risk assessment tool, BCRAT, breast cancer prediction, Gail model, Asia, and Asian*.

**Results:**

The search yielded 20 articles, with 7 articles addressing the AUC and/or the expected (E) to observed (O) ratio of predicted breast cancer risk, representing the accuracy of the GM in the Asian population. One publication reported the sensitivity and specificity but no AUC. None of the studies were accepted as the standard for reporting prognostic models. Several studies reported good prognostic testing and likely developed a new model modifying the items in the instrument.

**Conclusion:**

The results are not strong enough to develop breast cancer risk in the setting of Asian countries. Involving the breast cancer risk of the Asian population in developing a prognostic model with good statistical understanding is particularly important and can reduce flawed or biased models. Identifying the best methods to achieve well-suited prognostic models in the Asian population should be a priority.

## 1 Introduction

Breast cancer is the second most common cancer worldwide and is the highest leading cause of cancer-associated death among women worldwide. Both the incidence and mortality of breast cancer vary among populations throughout the world; it is estimated that over a half of new cancer cases diagnosed among women are in developing countries. In 2018, according to GLOBOCAN, newly diagnosed cases and breast cancer-associated deaths accounted for approximately 11.6% and 6.6% of all cancer types, respectively [1]. This trend has been growing even in Asian developing countries in recent years [2, 3, 4]. The increased incidence of breast cancer is especially seen in middle-income countries due to lifestyle changes, growing urbanization, changes in reproductive and dietary patterns, obesity, smoking, drinking alcohol, and reduced exercise [5, 6]. In addition, the mortality of breast cancer in these countries is generally higher than that in Western countries due to the limitations of health care settings and resources for breast cancer screening, as those in Asian countries [7, 8, 9]. Although high-income Asian countries such as Israel, Kuwait, Qatar, the Republic of Korea, Singapore and the United Arab Emirates have adequate health care services, most people living in many low-income Asian countries have limited health services and a substantial burden of cancer compared with other diseases. Therefore, increasing awareness and identifying risk factors are crucial for the prevention of breast cancer and for screening programs that aim to reduce the incidence of breast cancer. Women who have increased awareness of the early symptoms of breast cancer (if there is a change in their breasts) will immediately conduct an early health check. The early diagnosis of breast cancer is one of the best approaches to prevent this disease [10]. Insufficient knowledge about the risk factors and early symptoms of cancer is significantly associated with the majority of breast cancer patients diagnosed at an advanced stage, especially in developing countries, including Asia [11].

The Breast Cancer Risk Assessment Tool (BCRAT), also known as the Gail model (GM) (available at http://www.cancer.gov/bcrisktool/), is the most commonly used to predict breast cancer risk and was originally developed for use in white females to estimate breast cancer risk [12]. This model was originally developed for use in the US [13, 14, 15, 16]. To date, the GM has been widely recognized and adapted for specific ethnics populations in the US such as White-American [17, 18], Asian and Pacific Islander populations [19], and African-American [20, 21] populations, representing a wide range of study populations, health care settings, and sampling designs. However, the GM actually mentions the prediction of breast cancer risk in Americans among its items, reducing its usefulness outside the US setting. Indeed, a comparison of these studies suggests differences in the relative importance of the individual breast cancer risk, and these differences may result from disparities in the various racial and ethnic groups, considering diversity in epidemiology and the risk factors of breast cancer in populations such as Asian females [22, 23]. Consequently, the application of the GM has varied across studies, as evidenced by the different numbers and natures of the risk factors generated [24, 25, 26, 27, 28, 29, 30, 31, 32, 33, 34, 35, 36, 37, 38, 39, 40, 41, 42, 43]. Although many studies have examined and applied the GM, its use has been questionable particularly in Asian females. Based on the main concern about the effectiveness of the application of the risk assessment tool for developing breast cancer, especially in the Asian context, a systematic review to summarize all available evidence from the study population among Asian females is needed, particularly in middle-income countries, where racial, ethnic, religious and inadequate health care settings contribute to the risk of breast cancer is needed. Adequate knowledge about breast cancer risk factors in Asian populations is expected to reduce breast cancer mortality, especially in Asian countries.

## 2 Material and methods

**Figure 1:**
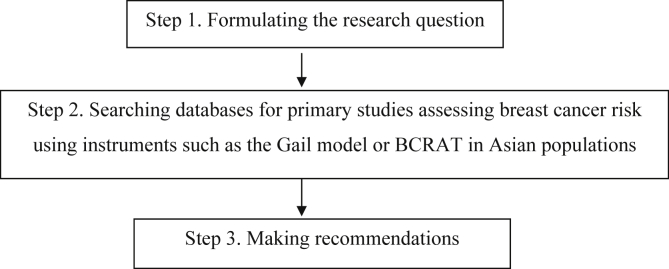
Three steps of the research process.

As shown in Figure 1, this three-step study was designed to evaluate the outcomes of the risk of breast cancer using the GM or BCRAT in an Asian population. We followed the PIOT/PICO (P – Population, I – Issues, O/C – Outcome/Comparison, T – Type of study) framework to answer the research question. The PIOT/PICO model is a tool used to organize and focus database queries to help identify terms and concepts in the literature search [44]. The researcher modified the model as a guide for answering the research questions, as illustrated in Table 1.

**Table 1:** Research question based on the PIOT model.

PIOT component	
Population (P)	Breast cancer patients and women in the Asian population
Issues (I)	Application of a breast cancer risk instrument in the Asian population
Outcome (O)	Reporting on the diagnostic breast cancer risk using the Gail model
Type of study (T)	Cross sectional, retrospective, and cohort

∗P = Population; I = Issue; O = Outcome; T = Type of Study.

The first step involves formulating the research question, thereby conducting a systematic literature search within the Asian context. The following PIOT question has been developed for this current study: What are the views of performance for GM to predict breast cancer risk in Asia countries? The last step consists of making recommendations for breast cancer instruments using the GM for Asian populations.

### 2.1 Search strategy in databases about instrument-risk breast cancer

Comprehensive keyword searches in databases such as PubMed, MEDLINE, Scopus, Web of Science (Science Citation Index (SCI) and Social Science Citation Index (SSCI)), and Google Scholar as well as gray literature sources were considered to identify breast cancer risk using the GM applied in the Asian population. The last electronic search was conducted on June 19, 2019. The main keywords were entered by a combination of Medical Subject Heading (MESH) terms and text words, including “breast cancer” OR “mammary” OR “carcinoma” OR “tumor” OR “neoplasm” AND “risk assessment tool” OR “BCRAT” OR “breast cancer prediction”, “Gail model”, and “Asia” OR “Asian”. Any publication of every design (observational studies, cross-sectional, cohort, case studies, case series, clinical trials, etc.) were identified and searched from January and May 2019. Studies that met the following inclusion criteria were included: published in English; accessible in full-text; assessed breast cancer risk instruments using the GM applied in Asian populations; and provided sufficient data. Sufficient data assessed by the method of all articles involving in this study addressed the area under the curve (AUC) or the expected (E) to observed (O) ratio of predicting breast cancer risk or measuring a 5-years breast cancer risk and lifetime breast cancer risk. The exclusion criteria were articles that were not published in English, including proceedings, case reports, scientific conference articles, article reviews, publications that were not in the databases above, and studies that did not report sufficient data.

## 3 Results

**Figure 2:**
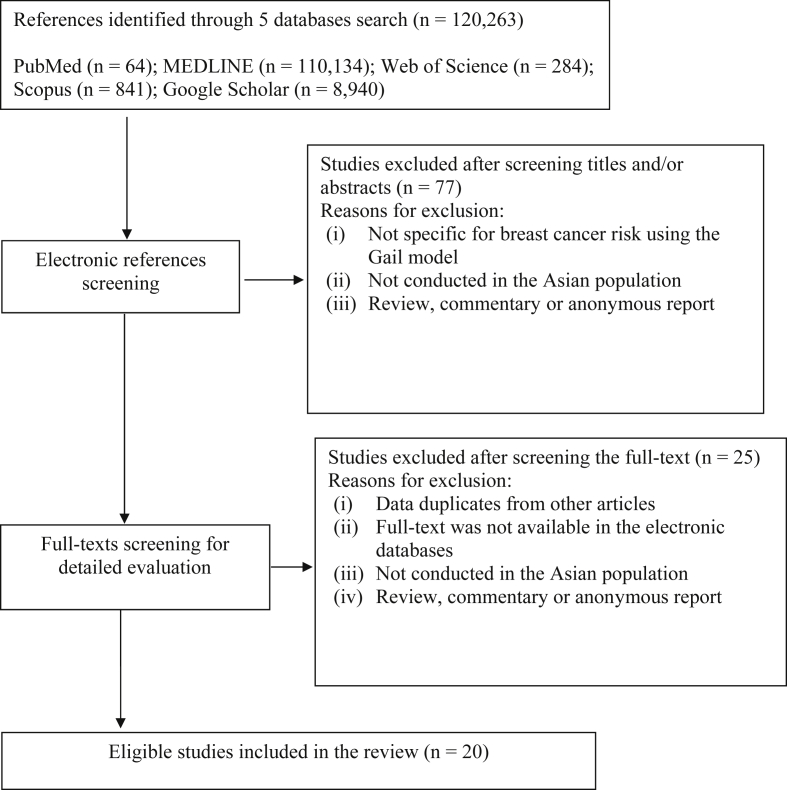
Flow diagram to illustrating the study selection procedure.

In total, 120,263 English language articles were retrieved starting from 2010 which was the year that the GM or BCRAT in the Asian population was available for testing, to 2018. There were 77 references found after a detailed screening of the titles and abstracts based on data related to the application of the breast cancer GM. Then, after full-text reviews and the removal of duplicated articles, as many as 25 articles were screened that further met the eligibility criteria. Five studies were discarded due to no available full-text report. Ultimately, only twenty relevant articles were used in this literature review (Figure 2).

Twenty articles were yielded from the initial search [19, 22, 23, 25, 29, 32, 38, 45, 46, 47, 48, 49, 50, 51, 52, 53, 54, 55, 56, 57]. Of these, seven articles specifically addressed the area under curve (AUC) and/or the expected (E) to observed (O) ratio of predicted breast cancer risk, which represented the accuracy of the GM in the Asian population [19, 29, 32, 38, 48, 54, 57]. One publication reported the sensitivity and specificity; however, an AUC was not yielded [45]. Twelve articles addressed the primary outcome, which was the follow-up of patients after a diagnosis of breast cancer [22, 23, 25, 46, 47, 49, 50, 51, 52, 53, 55, 56].

Of the publications that employed the GM in Asian populations, one was a longitudinal cohort study, two were retrospectively designed, two had prospective longitudinal formats, and five were observational case-control studies. In this systematic review, we included ten cross-sectional studies, one cohort study, one case control study and one prospective study because they focused on the follow-up of invasive breast cancer from the instrument application used. Based on the GM, those articles reported that the mean breast cancer risk at the five years and over a lifetime were uncertain. The characteristics of each article are summarized in Table 2 and 3.

**Table 2:** Notable publications in detail.

Study type	Total number of studies	Publication details
Cohort study	1	Park et al. [50]
Prospective study	2	Chay et al. [32], Zhao et al [54]
Case-control study	5	Matsuno et al [19], Gao et al [29], Challa et al [38], Min et al [45], Ulusoy et al [48]
Retrospective study	2	Thomas et al [25], Zhang et al [57]
Cross-sectional study	10	Yilmaz et al [46], Seyednoori et al [47], (Ceber et al [49], Erbil et al [51], Mohammadbeigi et al [52], Khazaee-Pool et al [22], Bener et al [23], Mirghafourvand et al [53], Ewaid and Al-Azzawi [55], Al Otaibi [56]

**Table 3:** Summary table of reviewed articles.

Reference	Country	Year of publication	Design	Cases	Age, years	Study population	Sensitivity, specificity, AUC	5-year breast cancer risk	Lifetime breast cancer risk	The expected (E) to observed (O) ratio for predicted breast cancer risk
Gail et al [12]	USA	1989	Case-control study	4496	>50	White females in the Breast Cancer Detection Demonstration Project (BCDDP)	-	1.02	11.21	-
Ulusoy et al [45]	Turkey	2010	Case-control	650	>35	Turkish females	Sensitivity = 13.3% Specificity = 92%, AUC: -	1.67	7.70	-
Yilmaz et al [46]	Turkey	2011	Cross-sectional	415	>20	Turkish population	-	1.7%	15%	-
Seyednoori et al [47]	Iran	2012	Cross-sectional	314	≥35	Iranian Women	-	0.80 (SD ± 1)	9.0 (SD ± 3.9)	-
Matsuno et al [19] (the modified Gail instrument)			Case-control study	1541	20–55	Asian-Americans in the Women's Health Initiative	AUC = 0.614, 95% CI: 0.587, 0.640	-	-	1.17, 95% CI: 0.99, 1.38
Chay et al [32]	Singapore	2012	Prospective study	28,104	50 to 64	The Singapore Breast Cancer Screening Project (SBCSP)	-	-	-	2.51 95% CI: 2.14, 2.96
Gao et al [29]	Singapore		Nested case-control study	28,883	≥45	The Singapore Breast Screening Program	AUC = 0.6098, 95% CI: 0.57, 0.65	-	-	1.00 95% CI: 0.88, 1.14
Challa et al [48]	India	2013	Case-control	200	>35	Indian population	Sensitivity = 51.9%; Specificity = 64%; AUC = 0.543	-	-	-
Ceber et al [49]	Turkey	2013	Cross-sectional	4,815	≥50	Turkish females	-	17.6%	0.2%	-
Park et al [50] (The modified Gail model)	Korea	2013	Cohort	3,789	49.0 ± 9.47 years	Seoul Breast Cancer Study	-	Case: 0.442 (SD = 0.148); Control: 0.450 (SD = 0.142)	Case: 2.241 (SD = 0.957); Control: 2.266 (SD = 0.941)	-
Min et al [38]	Korea	2014	Case-control	40,229		The Korean Breast Cancer Registration Program	AUC = 0.547, 95% CI: 0.500, 0.594	-	-	2.46 95% CI: 2.10, 2.8
Erbil et al [51]	Turkey	2015	Cross-sectional	231	>35	Turkish women	-	0.88 ± 0.91%	9.3 ± 5.2%	-
Mohammadbeigi et al [52]	Iran	2015	Cross-sectional	296	>34, 47.8 ± 8.8	Iranian females	-	0.37 ± 0.18	4.48 ± 0.925	-
Khazaee-Pool et al [22]	Iran	2016	Cross-sectional	3,847	>35	Iranian women	-	11.71 ± 3.91%	-	-
Bener et al [23]	Qatar	2017	Cross-sectional	1488	≥35 (47.8 ± 10.8)	Arabic women	-	1.12 ± 0.52	10.57 ± 3.1	-
Thomas et al [25]	India	2016	Retrospective study	222	>20	Indian population	-	92%	86%	-
Mirghafourvand et al [53]	Iran	2016	Cross-sectional	560	≥35	Iranian population	-	0.6% (SD = 0.2%)	8.9% (SD = 2.5%)	-
Zhao et al [54]	China	2017	Prospective study	3030	45–70	Chinese females	Sensitivity = 5% Specificity = 97.1% AUC = 0.542, 95% CI: 0.426, 0.658			
Ewaid and Al-Azzawi [55]	Iraq	2016	Cross-sectional	250	≥35	Iraqi population	-	11.30 ± 4.5%	-	-
Al Otaibi [56]	Saudi Arabia	2017	Cross-sectional	180	≥35, 41 ± 7.2	Saudi females	-	9.6 ± 5.4	-	-
Zhang et al [57]	China	2018	Retrospective study	280	35–69	Chinese population	Sensitivity = 53.33% Specificity = 77.69% AUC = 0.665, 95% CI: 0.629, 0.701	-	-	-

## 4 Discussion

This review highlights the scarcity of studies that have investigated the prediction of breast cancer risk using instruments such as the GM, especially focusing on Asian populations, with a detailed appraisal of the characteristics of model performance, such as calibration, discrimination and accuracy. In particular, 6 studies provided an evaluation of how successful their prognostic models were, while most studies had no validation at all. However, none of the instruments in our literature review that have been validated were reported to be unsuitable with the standard of prediction models.

Instruments that have good calibration show a good discriminative capacity of the model to separate patients who experience events from those who do not [58, 59, 60, 61]. The standard of the discrimination test can be presented by a Kaplan-Meier graph from a survival analysis with different risk groups of breast cancer. Several tests of discrimination are provided by the R square value or the goodness of fit model [60], D statistic [62], c-index [60], the net reclassification improvement (NRI) [63, 64], the integrated discrimination improvement (IDI) [63], decision curve analysis [65], separation (SEP) and the prognostic separation index (PSEP) [66, 67]. Categorical variables in predictive models can be examined by a comparison of the risk groups for breast cancer (for example, log rank and NRI), while continuous variables can be applied by only one of the tools, such as the c-index or D statistic. In this article, we found that none of the studies had an accepted standard of reporting for prognostic models, particularly in addressing Asian populations [68, 69]. However, several items in this instrument reported good prognostic testing, and it is likely that these items were conducted as a new model that was developed in some studies.

A good performance was mostly reported for the GM as a prognostic model among Western populations, such as American [70], Canadian [71], British [72], and Swedish populations bib73[73, 74, 75, 76]. In our study, two publications applied the GM in Asian populations, such as Turkish [45] and Singaporean populations [32]; however, they had uncertain results in predicting invasive breast cancer, particularly among Asian populations. In addition, when the 5-year risk of 1.67% was employed as the cut-off point for the definition of high risk, several studies revealed that the current GM is inadequate for predicting individualized breast cancer risk among Asian women [22, 23, 25, 45, 46, 47, 49, 50, 51, 52, 53, 55, 56]. The primary reason for the inadequate prediction of breast cancer risk using the GM is multifactorial, including varied ethnicity among breast cancer groups, patient characteristics, lifestyle changes and population aging.

Some limitations should also be acknowledged. First, several studies evaluated in this current study did not utilize the standard tools for assessing the methodological quality of the studies conducting prognostic testing. This is because a limited number of studies in Asian women and published in English have employed predicting breast cancer risk using the GM model Second, some randomized trials followed up patients with invasive breast cancer, whereas prospective studies involved in this literature review were rare. However, our literature review had some strengths. First, a total of 20 published studies were not limited to publications with cross-table data but extended to studies with AUCs and 95% CIs, the expected (E) to observed (O) ratio or the lifetime or 5-year follow-up of breast cancer risk. Second, the sample size conducted in the literature review was sufficient to estimate the reliability and enhance the statistical power of the data analysis. Third, the included studies were conducted in different countries, which made the results more generalizable. Therefore, we concluded that the results based on the current evidence are relatively convincing.

## 5 Conclusions

In general, the current study has provided evidence that the application of the GM in predicting breast cancer risk among the Asian population is scarce. The results are not strong enough to develop breast cancer risk in the setting of Asian countries. At present, there is a paucity of adequate performance of the GM in Asian countries for the model to be applicable across cultures or even outside the health care setting in which such instruments were developed. Involving the breast cancer risk of the Asian population in the development of a prognostic model with good statistical understanding is particularly important and can reduce flawed or biased models. Further research is necessary to identify the best methods to achieve well-suited prognostic models in the Asian population and should be a priority.

## Declarations

### Author contribution statement

All authors listed have significantly contributed to the development and the writing of this article.

### Funding statement

This work was supported by Kemenristek Dikti (Ministry of Research, Technology and Higher Education of Republic Indonesia) No PD-016/SKPP.TJ/LPPM UAD/III/2019.

### Competing interest statement

The authors declare no conflict of interest.

### Additional information

No additional information is available for this paper.
